# Heyde syndrome secondary to hypertrophic obstructive cardiomyopathy: a case report

**DOI:** 10.1186/s43044-026-00727-6

**Published:** 2026-03-09

**Authors:** Abraham Castellanos-Maldonado, Gustavo Abraham Canales-Azcona, José Luis Martínez-Arroyo, Miguel Maldonado-Valdéz, Héctor Raúl Ibarra-Sifuentes

**Affiliations:** Escuela de Medicina Unidad Norte, Universidad Autónoma de Coahuila, Piedras Negras, Coahuila, Mexico

**Keywords:** Heyde syndrome, Hypertrophic obstructive cardiomyopathy, Gastrointestinal bleeding, Transcatheter aortic valve replacement, Case report

## Abstract

**Background:**

Heyde syndrome is an uncommon clinical condition characterized by the triad of aortic stenosis, gastrointestinal angiodysplasia, and acquired von Willebrand syndrome. Its occurrence in the context of hypertrophic obstructive cardiomyopathy is rare. Common in individuals over 65, diagnosis is challenging due to the prevalence of these conditions. Hypertrophic obstructive cardiomyopathy increases shear stress, leading to von Willebrand Factor degradation and a higher risk of gastrointestinal bleeding.

**Case presentation:**

A 69-year-old male presented to the outpatient clinic with melena, abdominal cramping, and fatigue, along with a history of anemia requiring blood transfusions. Despite a negative upper gastrointestinal endoscopy, further tests revealed angiodysplasias with recent bleeding. He was also diagnosed with hypertrophic cardiomyopathy with left ventricular outflow tract obstruction. After being treated with octreotide and nadolol, his condition improved markedly, with hemoglobin levels rising and no further episodes of bleeding.

**Conclusion:**

Recognizing hypertrophic obstructive cardiomyopathy as a new variant of Heyde syndrome broadens our understanding of this complex disorder. It emphasizes the need to consider hypertrophic obstructive cardiomyopathy in patients with unexplained gastrointestinal bleeding and cardiac murmurs, prompting a more tailored approach to diagnosis and treatment.

## Background

Heyde syndrome (HS) is a multisystem disorder characterized by aortic stenosis (AS) and recently hypertrophic obstructive cardiomyopathy (HOCM), gastrointestinal angiodysplasia (GIAD), and acquired von Willebrand syndrome (AVWS). The exact prevalence and incidence of HS are currently unknown; however, it is likely to be an underdiagnosed and underreported clinical entity from what the literature reflects. HS is frequently diagnosed in persons older than 65 years, a population in which there is also a high prevalence of AS and GIAD, making the diagnosis of HS difficult [1]. More recently Batur P, et al. revealed a 31.7% prevalence of AS among patients with gastrointestinal (GI) arteriovenous malformations [2].

HOCM is an inherited heart condition characterized by cardiac hypertrophy independent of abnormal heart workload, along with left ventricular obstruction of ≥ 15 mmHg. HOCM occurs when hypertrophy of the interventricular septum and systolic anterior movement (SAM) of the mitral valve leaflet results in the left ventricular outflow tract (LVOT) obstruction [3]. HOCM affects about 0.16% to 0.29% of the adult population without any specific geographic, ethnic, or sex distribution [4].

GIADs are vascular malformations composed of dilated blood vessels located in the mucosa and submucosa of the GI tract. GIADs are increasingly identified as a cause of recurrent small intestine GI bleeding (formerly known as obscure GI bleeding) and iron-deficiency anemia. GIADs are incidentally found in 1% to 5% of patients undergoing endoscopic studies, they are typically observed in individuals over 60 years old, suggesting a degenerative aspect in their development [5–7].

Recently, AVWS has been increasingly recognized as a condition affecting individuals with various hematological, endocrine, and cardiovascular disorders. It involves alterations in the quantity, structure, or function of von Willebrand Factor (vWF), leading to abnormal clotting mechanisms [8]. The increased prevalence of AVWS may be due to the aging population and conditions like AS, mechanical circulatory support, and other etiologies involving shear forces [9]. All in all, HOCM increases shear stress on blood flow, degrading vWF and leading to AVWS. This impaired clotting heightens the risk of bleeding in GIAD, making patients with HOCM susceptible to significant GI bleeding.

Herein, we report a rare case of small intestinal angiodysplasia with concomitant HOCM. To the best of our knowledge, only about 10 similar cases have been described in the literature. This article aims to identify HOCM as a novel variant of HS, review the mechanisms responsible for bleeding in such cases, and provide insights into its clinical implications.

## Case presentation

A 69-year-old male presented to the gastroenterology outpatient clinic, with complaints of “dark, tarry stools” and abdominal cramping that began the previous day. He also reported new-onset mild shortness of breath on exertion and significant fatigue during the same period. Relevant past medical history included heavy smoking and moderate alcohol consumption. The patient had experienced multiple hospital admissions for anemia, including a recent episode of grade IV microcytic, hypochromic anemia with a hemoglobin (Hb) level of 5.5 g/dL (Table 1). He received two units of blood at that time, with a subsequent rise in Hb to 8.8 g/dL approximately one week later. Despite undergoing an upper GI endoscopy about two weeks before the current presentation, which was negative for bleeding, his Hb level again decreased to 7.1 g/dL shortly thereafter. A few days before his current visit, he developed symptoms of low cardiac output, including dyspnea and dizziness, leading to another hospitalization and an additional blood transfusion.

**Table 1:** First complete blood count and basic metabolic panel performed

Complete blood count	Initial	Unit	Reference range
Complete blood Count			
WBC	9.28	10^3^/mm^3^	4.40–10.67
RBC	2.44	10^6^/mm^3^	3.50–5.80
Hemoglobin	5.5	g/dL	12.0–18.0
Platelets	424.2	10^3^/mm^3^	150.0–4500
Basic Metabolic Panel			
BUN	39	mg/dL	7.0–25.0
Glucose	94	mg/dL	69–105
Urea	32.10	mg/dL	2.33–46.70
Creatinine	0.94	mg/dL	0.70–1.20
Sodium	138.0	mEq/L	136–145
Potassium	4.3	mEq/L	3.5–5.1
Chloride	111.0	mEq/L	98.0–107.0
Liver Function Test			
ALT	12.00	U/L	7.00–52.00
AST	16.00	U/L	14.00–78.00
Total bilirubin	0.5	mg/dL	0.22–1.04
Direct bilirubin	0.2	mg/dL	0.0-0.2
Indirect bilirubin	0.1	mg/dL	0.1–0.8
Alkaline phosphatase	51.00	U/L	34–104
LDH	184	U/L	105–333
INR	0.98	U/L	0.9–1.80

WBC, white blood cells; RBC, red blood cells; BUN, blood urea nitrogen; ALT, alanine aminotransferase; AST, aspartate aminotransferase; LDH, lactate dehydrogenase; INR, international normalized ratio

**Fig. 1:**
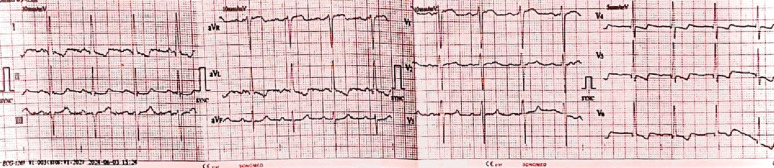
The ECG findings with features indicative of left ventricular hypertrophy

**Fig. 2:**
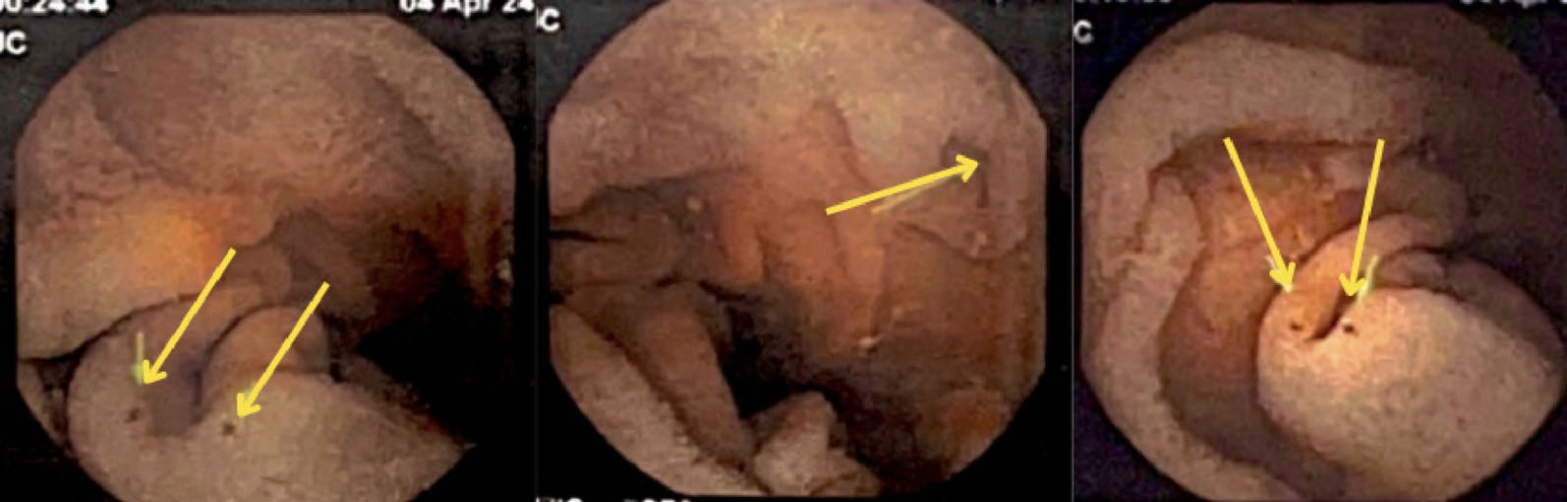
Small and medium-sized angiodysplasias in the distal duodenum and proximal jejunum, with signs of recent bleeding

On physical examination, the patient was hemodynamically stable, with a blood pressure of 122/86 mmHg, heart rate of 90 beats per minute, respiratory rate of 22 breaths per minute, temperature of 36.5 °C, and an oxygen saturation of 97%. He appeared pale with cold extremities and weak peripheral pulses, though no peripheral edema was noted. His chest examination revealed normal breath sounds bilaterally, though a crescendo-decrescendo systolic murmur between the apex and left sternal border was audible. The rest of the physical examination was unremarkable. Laboratory investigations showed a hemoglobin level of 7.2 g/dL, hematocrit 24.1%, white blood cell count of 8.7 × 10^3/mm3, and platelet count of 508 × 10^3/mm3. An electrocardiogram showed evidence of left ventricular hypertrophy (Fig. 1). A colonoscopy was performed, which revealed a 4 mm sessile polyp in the sigmoid colon, while an abdominal doppler ultrasound showed only fatty liver. Subsequently, a capsule endoscopy, identified angiodysplasias in the distal duodenum and proximal jejunum. (Fig. 2).

**Fig. 3:**
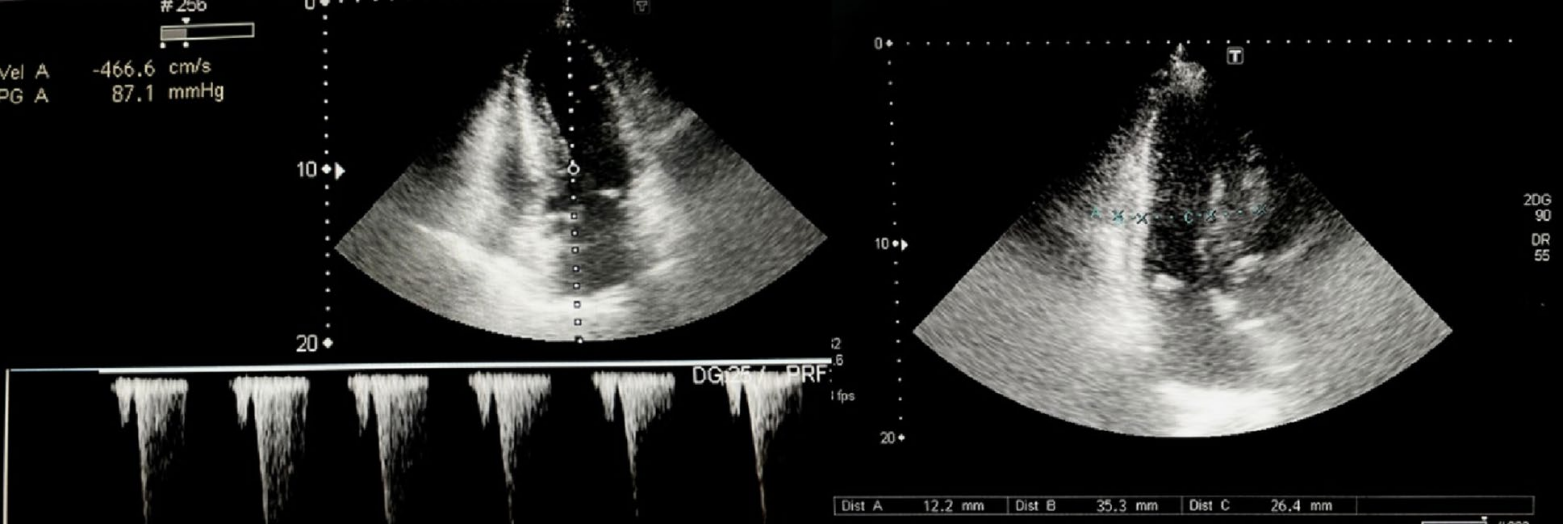
Transthoracic echocardiogram revealed HOCM, septal hypertrophy of 26.4 mm, posterior wall thickness of 12.2 mm, dynamic obstruction of the LVOT, with a maximum velocity of 4.6 m/s and a gradient of 87.1 mmHg. The left ventricular diastolic dimension was 35.3 mm, with a left ventricular ejection fraction (LVEF) of 70%. Mild diastolic dysfunction type 1 was present, along with mild left atrial dilation and no evidence of pulmonary hypertension

The patient was referred to the cardiology outpatient clinic due to the detection of a systolic murmur. A transthoracic echocardiogram was performed and revealed HCMO with SAM. (Fig. 3).

The patient was diagnosed with small-bowel GIADs and HCMO with dynamic LVOT obstruction. The association of these two conditions led to a diagnosis of a variant of HS. The patient was started on octreotide 50 mcg subcutaneously every 12 h for 3 months to manage the GIAD. For the management of obstructive asymmetrical septal HCM, nadolol was initiated at a dose of 20 mg daily to improve cardiac function and control symptoms.

At follow-up, the patient showed significant clinical improvement with no evidence of active bleeding and a Hb level of 8.2 g/dL. On subsequent follow-up, his hemoglobin level had risen to 12.3 g/dL, with no recurrence of melena episodes and continued improvement.

## Discussion

HS involves multifactorial bleeding mechanisms, prominently featuring AVWS. vWF, crucial for hemostasis, is synthesized by endothelial cells and megakaryocytes in large multimeric forms. In HS, high shear stress due to calcified and stenotic aortic valves cause fragmentation of large vWF polymers, mediated by hTS13, resulting in diminished high-molecular-weight (Hmw) vWF. This impairs platelet adhesion and aggregation, evidenced by reduced Hmw vWF levels and altered platelet function in severe AVS. Concurrently, GIADs occur, characterized by mucosal vascular lesions driven by chronic ischemia and VEGF-mediated angiogenesis. Reduced vWF levels exacerbate GIADs development, increasing shear stress and predisposing HS patients with AVS to GI bleeding [10].

HOCM is a condition caused by mutations in sarcomeric protein genes, where thickening of the heart muscle, particularly the interventricular septum, obstructs blood flow from the left ventricle to the aorta. It is one of the most common hereditary cardiomyopathies, affecting approximately 1 in 500 individuals. Despite its prevalence, the co-occurrence of HOCM with GIAD in the context of HS is infrequent, making such cases notable due to their rarity and clinical significance. Obstruction in HOCM arises from septal thickening and SAM of the mitral valve, where the mitral valve leaflets are pulled towards the septum during systole, causing mitral-septal contact. This phenomenon is driven by two primary mechanisms: the Venturi effect, in which accelerated blood flow through the LVOT creates negative pressure that pulls the mitral valve towards the septum, and the forceful ejection of blood against an elongated, distorted mitral valve, leading to leaflet displacement into the LVOT and potentially secondary mitral regurgitation. These mechanisms result in high-pressure gradients that degrade vWF multimers, potentially causing AVWS and related bleeding tendencies, such as GIADs [3, 11]. On the other hand, in patients with AS, the constricted valve causes high-velocity blood flow, increasing shear stress on vWF multimers, particularly the Hmw, ultimately predisposing patients to spontaneous GI bleeding [1]. Many patients with HOCM are asymptomatic, however, in symptomatic patients the most common clinical manifestations include LVOT gradients, dyspnea, fatigue, chest pain, and syncope. During physical examination, a systolic ejection murmur is commonly detected during auscultation and increases with maneuvers that decrease preload and afterload. In adults, 2D-echocardiography is the primary diagnosis tool, with an 80% accuracy for HOCM. Key features include asymmetric septal wall thickness (> 15 mm), SAM of the mitral valve leaflet, and noticeable LVOT narrowing. Patients with suspected HOCM who have normal, or inconclusive echocardiogram results should undergo cardiac magnetic resonance imaging, as it is the gold standard for diagnosing LV wall properties [12]. Medical therapy is the primary approach for managing symptoms in HOCM. Non-vasodilating β-blockers are typically the first-choice treatment for HOCM, the primary goal is to relieve HCM symptoms by effectively slowing the heart rate. They should be titrated to achieve a resting heart rate of 50–60 beats per minute. Patients who remain symptomatic despite taking the appropriate drug therapy and who are at high risk of sudden cardiac death are candidates for septal relief therapy, being surgical myectomy the gold standard for these patients [3]. According to a prospective cohort study, remission of GI bleeding was observed in 85% of patients after septal myectomy [13]. Wake et al. and previous case reports have demonstrated that medical therapy can be effective in selected cases of HOCM associated GI. In these cases, medical therapy (non-vasodilating β-blockers and calcium channel antagonist) were enough to resolve bleeding episodes [14]. In contrast, Blackshear et al. reported that patients with severe obstruction, or those with recurrent bleeding despite medical and endoscopic therapy benefited from septal reduction procedures [15].

GIADs can occur throughout the digestive tract; recent studies using video capsule endoscopy and device-assisted enteroscopy have shown they are most common in the small bowel (57%-80%), followed by the colon (44%) and stomach (32%). They account for 4% to 7% of nonvariceal upper GI bleeding cases [16]. The clinical presentation of bleeding from GIADs varies from occult bleeding to severe hemorrhage necessitating large transfusions and emergency intervention. Spontaneous cessation of bleeding occurs in approximately 45–50% of cases; however, rebleeding affects about 34% of patients (45% in small-bowel), with 16–64% experiencing recurrent anemia or requiring transfusions within 1 to 3 years [17]. Treatment for bleeding GIADs includes endoscopic therapies, angiography with embolization, surgical resection, and pharmacologic therapy. While endoscopic therapy is initially effective, Jackson CS, et al. found high rebleeding rates, with an overall rate of 34% and 45% specifically for small bowel GIADs. However, given that patients are often elderly with comorbidities, endoscopic options may not always be optimal. Medical therapy for GIAD includes the use of somatostatin analogues and thalidomide. Somatostatin analogues, such as octreotide and lanreotide, work by inhibiting angiogenesis, reducing blood flow, and enhancing platelet aggregation. Studies have shown these analogues can reduce rebleeding rates with a significant reduction in recurrence and transfusion needs [17, 18].

The pathophysiology of AVWS involves three main mechanisms: (1) development of autoantibodies that impair and clear vWF, (2) adsorption of vWF on cancer cells increasing its clearance, and (3) loss of Hmw vWF multimers due to high shear stress or proteolysis. In specific cases such as AS, high shear stress can cause platelet activation and adsorption of vWF, while in other conditions like myeloproliferative neoplasms, proteolysis may be induced by calcium released from platelets [9]. The actual prevalence of AVWS in the general population remains uncertain due to a lack of large prospective studies, likely underestimation by physicians, and a significant recent increase in cases associated with cardiovascular disorders. In most cases, with approximately 80% of patients experiencing active bleeding, and recurrent bleeding occurring in about 20–33% of patients, particularly after major trauma or surgery. Diagnosing AVWS involves tests like those for von Willebrand disease, such as bleeding time, PFA-100, FVIII, vWF, and platelet-dependent vWF activities. Reduced activity/antigen ratios and loss of Hmw multimers indicate defects. Elevated vWF propeptide suggests accelerated clearance. Autoantibodies, detected through mixing studies and ELISA, confirm immune-mediated AVWS. The treatment goals in AVWS are to control acute bleeding, prevent high-risk bleeding, and achieve long-term remission by addressing the underlying disorder. Desmopressin can be used for some patients, but its effects are short-lasting. High doses of vWF/FVIII concentrates are necessary for replacement therapy. Intravenous immunoglobulin is effective, especially in AVWS associated with IgG-MGUS [19].

Currently, there are no standardized guidelines or unified protocols for managing HS. The treatment approach can be effectively divided into two main components: addressing the underlying cause of the bleeding and managing the GI bleeding itself. Since HS is often associated with AS, the management of AS through valve replacement is crucial. Among the treatment options, Transcatheter Aortic Valve Replacement has demonstrated superior effectiveness in resolving recurrent GI bleeding compared to other therapies. The initial approach to GI bleeding involves standard resuscitative measures, including intravenous fluids and blood transfusions, to stabilize the patient before identifying the source of bleeding or performing endoscopic interventions [1, 20].

## Conclusions

In conclusion, the recognition of HOCM as a new variant of HS highlights the expanding understanding of this complex multisystem disorder. The identification of this new variant underscores the importance of considering HOCM in patients with unexplained GI bleeding and cardiac murmurs, as it broadens the spectrum of HS and calls for a tailored approach to diagnosis and management.

## Data Availability

No datasets were generated or analysed during the current study.

## Abbreviations

HS: Heyde syndrome
AS: Aortic stenosis
HOCM: Hypertrophic obstructive cardiomyopathy
LVOT: Left ventricular outflow tract
SAM: Systolic anterior movement
GIAD: Gastrointestinal angiodysplasia
AVWS: Acquired von Willebrand Syndrome
vWF: von Willebrand Factor
Hb: Hemoglobin
GI: Gastrointestinal
Hmw: High molecular weight
